# Relationships between Sensory Processing and Executive Functions in Children with Combined ASD and ADHD Compared to Typically Developing and Single Disorder Groups

**DOI:** 10.3390/brainsci14060566

**Published:** 2024-06-02

**Authors:** Zhi Huang, Fang Wang, Leran Xue, Huilin Zhu, Xiaobing Zou

**Affiliations:** Child Developmental-Behavioral Center, The Third Affiliated Hospital of Sun Yat-sen University, No. 600, Tianhe Rd., Guangzhou 510630, China

**Keywords:** autism spectrum disorders, attention-deficit/hyperactivity disorder, sensory processing, executive function, inhibition, children

## Abstract

The prevalence of autism spectrum disorder (ASD) and attention-deficit/hyperactivity disorder (ADHD) is increasing, with a tendency for co-occurrence. Some studies indicate a connection between atypical sensory processing and executive function. This study aims to explore the distinctive etiology of executive function deficits in children with ASD+ADHD by investigating the relationship between sensory processing and executive function, comparing children with ASD, ASD+ADHD, ADHD, and typically developing children (TD). Method: Sensory Profile 2 (SP-2) and Behavior Rating Inventory of Executive Function 2 (BRIEF-2) were measured in 120 school-aged children. The results of the above scales were compared across these four groups, and correlation and regression analyses between BRIEF2 and SP2 were conducted. Results: Our research revealed varying levels of atypical sensory processing and executive function anomalies across the three neurodevelopmental disorder groups compared to the TD group. The ASD+ADHD group showed particularly significant differences. The heightened emotional problems observed in ASD+ADHD children may be associated with more prominent atypical sensory processing. Variance analysis of inhibitory function revealed differences between ASD+ADHD and ADHD children, suggesting distinct etiological mechanisms for attention issues between ASD+ADHD and ADHD. Conclusions: ASD+ADHD represents a phenotype distinct from both ASD and ADHD. Special consideration should be given to interventions for children with ASD+ADHD. The results of this study may offer a new perspective on understanding the occurrence of ASD+ADHD and potential individualized intervention methods.

## 1. Introduction

Autism spectrum disorder (ASD) and attention-deficit/hyperactivity disorder (ADHD) are neurological developmental disorders resulting from the interaction of multiple genetic and complex environmental factors, often occurring concurrently [1]. DSM-5 updated the diagnostic criteria for ADHD and ASD, affirming the diagnosis of comorbidity between the two disorders [2]. However, prior to this revision, clinicians were unable to make a diagnosis of comorbid ADHD with ASD. The prevailing belief was that symptoms of ADHD were secondary to ASD, rather than indicating an additional ADHD diagnosis [3], resulting in relatively fewer studies on ASD+ADHD compared to ASD and ADHD. ADHD is the most prevalent comorbidity in ASD, with approximately 40–70% of people with ASD exhibiting comorbid ADHD or significant subthreshold symptoms [4]. Individuals with ASD+ADHD display more pronounced characteristics than those with ASD or ADHD alone, including heightened social and adaptive behavior disorders [5]. In comparison to individuals with ASD alone, children with ASD+ADHD exhibit a greater number of psychiatric symptoms, often requiring more psychological and pharmacological interventions [6]. Moreover, when compared to people with ADHD alone, individuals with ASD+ADHD often show reduced response to psychostimulants and are more prone to experience severe adverse reactions to medication side effects [7,8,9]. For individuals with ASD+ADHD, intervention combining ASD and ADHD therapies may be effective if they exhibit only typical symptoms of either disorder. However, if both disorders mutually influence each other, the treatment will be more complicated [10]. Is ASD+ADHD merely an aggregation of symptoms from ASD and ADHD, or does it represent a unique pathological phenotype? Exploration of the relationship among these three disorders may help identify the core issues of ASD+ADHD and provide clues for precision therapy.

### 1.1. Atypical Sensory Processing

Atypical sensory processing (SP) is observed in both ASD and ADHD. Sensory symptoms constitute the core diagnostic criteria of ASD. Up to 95% of individuals with ASD demonstrate atypical SP [11,12]. Additionally, 66% of children with ADHD also exhibit atypical SP [13]. ASD+ADHD manifests particularly pronounced atypical SP compared to the other two groups [14]. In Dunn’s sensory processing model, individuals’ responses to sensory stimuli are influenced by their neurological sensitivity threshold and corresponding response strategies. Within the framework of environmental stimulus processing, low registration is typified by a heightened detection threshold and the utilization of passive response strategies. Sensory seekers, characterized by high thresholds and active response strategies, stand in contrast to individuals exhibiting sensory sensitivity, typified by a low threshold and passive response strategies. Those exhibiting a low threshold and engaging in active response strategies are categorized as sensory avoiders [15]. Sensory avoidance is a predominant feature of ASD, while sensory seeking is more characteristic of ADHD [16]. The distinct sensory patterns of these two disorders may represent differing sensory thresholds and corresponding responding strategies. Atypical SP is considered to be correlated with the severity of the core characteristics of ASD and impact the social, cognitive, and adaptive functioning of individuals with ASD [11,17]. Atypical SP exacerbates behavioral problems in ADHD, correlating with inattention and working memory deficits [18,19].

### 1.2. Executive Function Deficits

Executive function (EF) deficits are commonly observed in individuals with ASD and ADHD. EFs are advanced cognitive processes, crucial for guiding and regulating emotions, actions, and attention [20,21]. Despite significant individual differences, ASD often presents with shift impairments, indicating a deficiency in cognitive flexibility, while ADHD typically exhibits deficits in working memory and inhibition functions [22]. Cognitive flexibility, defined as the ability to adjust to variations in the environment and to meet new demands effectively, is considered to be the most severely impaired component of executive function in individuals with ASD [23]. Individuals with ASD exhibit significant difficulties in adapting their behavior to various social environments [24]. They tend to adhere strongly to rigid routines and may experience uncontrollable temper outbursts in response to subtle environmental changes, which can significantly impact learning [25], social skills [26], adaptive abilities [27], and quality of life [28]. Nevertheless, the neural mechanisms that account for this impairment in both ASD and ASD+ADHD are still unclear. The capacity for inhibitory functions encompasses the ability to cease one’s own behavior at the appropriate moment, often identified as the primary deficit in ADHD, particularly the hyperactive/impulsive subtype [29,30]. Additionally, ASD+ADHD exhibits more impairment in inhibition compared to ADHD. Research comparing executive function (EF) in children with ASD, ADHD, and ASD+ADHD indicates that the EF deficits in individuals with comorbid symptoms are additive. Children presenting with both symptoms exhibit more EF deficits than those with a single diagnosis [31,32,33].

### 1.3. Sensory–Cognitive Regulation in Neurodevelopmental Disorders

Individuals with ADHD may exhibit abnormalities in autonomic nervous system function, i.e., reduced activation of the sympathetic nervous system, leading to difficulties in focusing attention, high sensory thresholds, and impaired executive functions. Significant sensory stimuli may sustain their arousal levels, and hyperactivity may be a compensatory manifestation of ADHD. Consequently, individuals with ADHD often seek stimulation and display heightened interest in sensory stimuli. Yet, excessive exploration of their surroundings may also divert their attention [34,35], resulting in deficits in inhibitory function within EF. Children with ASD frequently display excessive reactions to sensory stimuli and struggle to regulate their autonomic nervous system based on environmental demands. Consequently, they may encounter challenges in processing information efficiently in sensory-rich contexts, leading to sensory avoidance [36]. This could be attributed to reduced top-down control of the autonomic nervous system, leading to an excessive release of dopamine and norepinephrine, thereby heightening sensory sensitivity and initiating anxiety. Impairments in EF are compounded by these factors, given that emotional regulation is an indispensable component of EF. Are the EF issues in children with ASD+ADHD influenced by sensory–cognitive dysregulation or abnormal neurotransmitter secretion? There is currently no literature dedicated to elucidating this question. Investigating the relationship between sensory processing and EF in ASD+ADHD may shed light on the possible factors contributing to the various executive function deficits in comorbid individuals and consequently, allowing for the development of precise intervention plans.

### 1.4. Study Goals

This study aims to compare sensory processing and executive function characteristics across four groups (ASD, ADHD, ASD+ADHD, and TD), to analyze the correlation between SP and EF in each group, and to investigate the distinctive association between SP and EF in children with ASD+ADHD. Based on previous research findings, it is hypothesized that the atypical sensory processing and executive function deficits in the three neurodevelopmental disorder groups will be more pronounced compared to those in the TD group, with the ASD+ADHD group demonstrating the most prominent differences. The heightened and extensive atypical SP observed in the ASD+ADHD group might correlate with more severe executive function impairments.

## 2. Materials and Methods

### 2.1. Participants

A total of 120 participants, aged 7 to 14 years, were recruited for the study and were divided into four groups: ASD (*n* = 32), ASD+ADHD (*n* = 30), ADHD (*n* = 28), and TD (*n* = 30). Participants were recruited from the southern region of China through online recruitment advertisements, as well as from patients receiving treatment at the Child Development and Behavior Center of the Third Affiliated Hospital of Sun Yat-sen University. Eligible individuals were invited to participate in the clinical study. Preliminary online screening interviews were administered to registering parents to assess eligibility for the study and for group allocation. Individuals classified within the ASD group underwent evaluations conducted by developmental behavioral pediatricians to confirm the DSM-5 diagnosis of ASD, excluding comorbid ADHD. Recruitment occurred after meeting the threshold on both the Autism Diagnostic Interview-Revised (ADI-R) [37] and the Autism Diagnostic Observation Schedule (ADOS-2) [38]. The diagnostic procedure for enrolling participants in the ASD+ADHD group mirrored that of the ASD group, supplemented by the diagnosis of ADHD by pediatric developmental behavior specialists based on DSM-5 criteria. Participants in the ADHD group were recruited based on the DSM-5 criteria for ADHD, with assessments from the NICHQ Vanderbilt Rating Scale completed by teachers and parents, confirming the ADHD diagnosis and excluding those with concomitant diagnosis of ASD. The TD group was recruited from the community; individuals and their relatives in the TD group did not have any known neurological or psychiatric disorders in their background. The scores on the Social Responsiveness Scale and the NICHQ Vanderbilt Assessment Scale for screening ASD and ADHD did not reach clinical thresholds. After assessment by a developmental behavioral pediatrician, the qualifying children were enrolled. The participants reported no history of psychotropic medication use and had not received any pharmacological treatment for attention-related issues. Given the complexity of assessing EF in children with intellectual disabilities [39], participants with an overall IQ below 70 (determined by the Stanford–Binet, 5th Edition [40]), individuals with genetic syndromes, and children classified as “blind” or “hearing impaired” were excluded from the study.

### 2.2. Measures

The Child Sensory Profile-2 (CSP-2) is a questionnaire designed to assess sensory processing characteristics in children aged 3–14 [15]. It classifies sensory processing into four patterns, across six sensory systems. Within the CSP-2, parents provide ratings of their child’s sensory responses using a standardized scale. Scoring is based on raw scores, with lower scores indicating low reactivity to sensory stimuli and higher scores indicating high reactivity. The goal of this research is to analyze the correlation between sensory reactivity and EFs among different groups; thus, we selected four sensory quadrants for analysis. The CSP-2 was translated into Chinese by a native Chinese speaker. Back-translation was completed by a bilingual individual whose native language is English, but who is unfamiliar with the scale. The original English version and the back-translated version were compared by an independent translator. Cronbach’s α values ranging from 0.63 to 0.97 were observed for both the overall scale and its subscales in the Chinese version of the SP-2.

The Behavior Rating Inventory of Executive Function, Second Edition (BRIEF2) is a parent-rated questionnaire, developed for the purpose of assessing EFs in children between the ages of 5 and 18. The questionnaire consists of 63 items, divided into nine subscales and four indices (Behavior Regulation Index (BRI), Emotion Regulation Index (ERI), Cognitive Regulation Index (CRI), and Global Executive Composite (GEC)). Higher scores indicate more severe EF impairment [29]. The T-scores from this scale were included in the analysis. Considering the executive function profiles of ASD, ASD+ADHD, and ADHD [30,41], the inhibition and shift subscales were included in the analysis, along with the four composite indices. Cronbach’s α values ranging from 0.89 to 0.97 were found for both the global score and the subscales of the Chinese version of the BRIEF2, indicating satisfactory internal consistency.

The non-verbal cognitive level was assessed using the “perceptual reasoning” section of the Wechsler Intelligence Scale for Children-IV (WISC-IV) [38]. FSIQ evaluations were conducted for all participants using the WISC-IV for Children, with higher scores indicating greater intelligence [42].

The Social Responsiveness Scale (SRS) is a caregiver-reported questionnaire that quantitatively assesses ASD-related characteristics. Higher scores indicate a higher degree of ASD characteristics.

### 2.3. Statistics

All analyses were conducted using IBM SPSS 25.0. Descriptive statistics were calculated for each group. Cronbach’s α values were calculated to evaluate the internal consistency of the SP-2 and BRIEF-2. Parametric variables were compared between groups using ANOVA, whereas non-parametric variables were assessed using the Kruskal–Wallis test. Post hoc analyses were executed, utilizing Bonferroni correction to account for any significant between-group discrepancies observed. Analyzing the correlation between SP and EF within each group, multiple linear regression analyses were conducted with EF T-scores as the dependent variable and age, non-verbal IQ, and Sensory Processing Pattern Scores as the independent variables for the ASD, ASD+ADHD, and ADHD groups. Pearson correlation coefficients are provided, unless the data deviated from normal distribution, in which case, Spearman’s nonparametric measures are utilized. A stepwise backward-elimination multiple regression analysis was conducted to explore significant correlations. The adequacy of the fitted model was assessed using the adjusted R2. 

## 3. Results

There were no significant disparities in gender and age observed in intergroup comparisons (*p* > 0.05). The TD group demonstrated significantly elevated non-verbal IQ scores compared to both the ASD and ASD+ADHD groups (*p* < 0.05), with no notable distinctions observed among the three neurodevelopmental disorder groups. Table 1 displays the characteristics of the participants.

### 3.1. Atypical Sensory Processing 

Table 2 shows the descriptive statistics of EFs, SP outcomes, and group comparisons. All three neurodevelopmental disorder groups exhibited significantly atypical SP compared to the TD group (*p* < 0.05), with the ASD+ADHD group demonstrating particularly pronounced differences. Apart from sensitivity sensation, the ASD+ADHD group reported significantly more atypical SP than the ASD group across the other three sections. Furthermore, the ASD+ADHD group showed a higher incidence of avoidance compared to the ADHD group (*p* < 0.05). Overall, the ASD+ADHD group exhibits significantly more atypical SP compared to the ASD and ADHD groups.

### 3.2. Executive Function 

Table 2 illustrates that all three neurodevelopmental disorder groups exhibited significantly more problems than the TD group across all four indices, along with inhibitory and shifting functions. The ASD+ADHD group reported higher levels compared to the ASD group across all indices (BRI, ERI, CRI, and GEC) and two subscales (Inhibit, Shift) (*p* < 0.05). Additionally, significant differences were observed between the ASD+ADHD and ADHD groups in BRI and ERI. Moreover, there was a higher level of CRI observed in the ADHD group compared to the ASD group.

### 3.3. Correlational Analyses of SP-2 and BRIEF-2

The results reveal a significant association between sensory processing and executive functioning across BRI, ERI, and CRI in both the ASD and ASD+ADHD groups. In the ADHD group, the focus of association between SP and EF is primarily on the BRI and CRI, with fewer correlations observed in the ERI compared to the other two groups. Regarding the core deficit in EF in ASD children, specifically shift, the ASD group exhibits correlation solely with avoiding, while in the ASD+ADHD group, avoiding, sensitivity, and registration all demonstrate correlation with shift. As for the core deficit in EF of ADHD children, a correlation with inhibit is observed in all four quadrants (seeking, avoiding, sensitivity, and registration), while the ASD+ADHD group correlates with inhibit in the seeking, avoiding, and registration quadrants. This might suggest that the ASD+ADHD group exhibits a broader range of correlations related to “sensory processing-shift” compared to the ASD group, while the association between “sensory processing-inhibition” is not as pronounced as in the ADHD group. Correlational analyses are displayed in Table 3, Table 4 and Table 5.

### 3.4. Regression Models SP-2 and BRIEF-2

In Table 6, regression models are depicted (models with no significance are not displayed). The first regression models for all groups included only variables with the highest variance that significantly contributed to the model. In the second model, non-verbal IQ, age, and gender were incorporated.

The regression analysis indicates that in the ASD group, avoiding explains 28.2% of the variance in BRI and 36.4% of the variance in ERI. For the ASD+ADHD group, avoiding explains 50.2% of the variance in ERI. In the ADHD group, seeking accounts for 44.5% of the variance in BRI. Regarding “shift”, avoiding in the ASD+ADHD group can explain 28% of the variance, whereas no predictable sensory pattern was observed in the ASD group. As for inhibition, the sensory processing of the ASD+ADHD group cannot predict this behavior, whereas seeking in the ADHD group could explain 43.1% of the variance. Atypical SP seems to play a pivotal role in the manifestation of ASD traits among children with ASD+ADHD, yet it cannot predict the inhibition issues in the comorbid group.

## 4. Discussion

This study investigates the impact of sensory processing on executive function. The results indicate that the neurodevelopmental disorder group exhibits more atypical SP compared to the TD group. The ASD+ADHD group exhibits particularly significant atypical SP, consistent with earlier research findings [16]. Regarding EF, all three neurodevelopmental disorder groups were reported to have experienced more challenges compared to the TD group. The ASD+ADHD group displayed particularly extensive and significant deficits in EF, which is in line with the results of previous studies [43,44]. As for shift and inhibit, the ASD+ADHD group exhibited more pronounced impairments when compared to the group with a single disorder.

In this study, children with ASD+ADHD exhibited more severe sensory avoidance compared to those with ASD alone. Research conducted previously has shown that individuals with ASD+ADHD are more inclined to experience simultaneous emotional and psychiatric issues compared to those with ASD alone [10]. The sensory avoidance in the ASD+ADHD group explains the majority of the variance in emotional regulation, whereas in the ASD group, it explained only a smaller portion. Therefore, the increased sensory avoidance observed in the ASD+ADHD group may have a significant impact on the emotional regulation functioning of children with comorbidities. In regard to the primary EF deficits associated with ASD and ADHD, including shifting and inhibition, it was revealed that only sensory avoiding in the ASD+ADHD group could predict shifting function, with no sensory processing pattern predicting shifting observed in the ASD group. Shifting function is considered to be associated with repetitive and stereotyped behaviors in ASD [41]. Therefore, it is speculated that the more pronounced avoidance observed in children with ASD+ADHD may affect cognitive flexibility, consequently leading to more severe ASD symptoms.

As for the core EF deficit in ADHD, inhibitory function, sensory seeking, in the ADHD group can explain 43% of the variance, whereas SP in the ASD+ADHD group cannot predict inhibition. This implies that there is no notable correlation between SP and inhibition in the ASD+ADHD group, which may be attributed to the distinct mechanism underlying inhibition deficits in individuals with ASD and ADHD comorbidity in comparison to those with ADHD alone. Attention deficit is present in both individuals with comorbid ASD+ADHD and those with ADHD, with ASD potentially exhibiting stronger abilities in selective attention but experiencing difficulties in attention switching. In contrast, individuals with ADHD may encounter greater susceptibility to distractions during tasks and struggle to sustain attention for long durations. According to the locus coeruleus-norepinephrine (LC-NE) system dysfunction hypothesis, individuals with ADHD exhibit insufficient excitatory neurotransmitter secretion, resulting in attention deficits and an inability to sustain focus. As a compensatory mechanism, individuals with ADHD seek sensory stimulation to elevate levels of excitatory neurotransmitters, which in turn leads to distractibility. This study confirms that sensory seeking in children with ADHD can predict inhibitory function, which aligns with this hypothesis. Yet, attentional problem in children with ASD+ADHD might be predominantly attributed to challenges in top-down regulation. Attention is regulated in two directions: bottom-up (driven by sensation) and top-down (sensory processing regulated by higher cortical regions) [45]. Individuals with ASD are considered to have deficits in both top-down and bottom-up regulation [46,47]. Bottom-up regulation refers to the brain’s automatic response and processing of external sensory inputs. Sensory over-responsivity is frequently observed in individuals with ASD, and excessive attention to sensory stimuli can lead to difficulties in sustaining attention, referred to as bottom-up attention regulation deficits [48]. Top-down regulation refers to the brain’s ability to selectively process sensory information through self-control mechanisms. By identifying diverse stimuli, the brain reassigns the significance of sensory information, further discriminating between distracting stimuli and task-relevant stimuli, enabling the brain to respond more rapidly and accurately [49]. Children with ASD often become overwhelmed by sensory stimuli, making it difficult for them to selectively focus their attention on tasks by effectively inhibiting distractors. In this study, we demonstrate that sensory over-responsivity (avoidance) in the ASD group can predict inhibition, which is consistent with the results of previous research [48]. However, despite the pronounced sensory over-responsiveness observed in the ASD+ADHD group, its correlation with inhibitory functions was not significant. This is consistent with the findings of Dellapiazza et al. [16], although they suggested that the study could not establish a predictive model due to sample size issues. Therefore, the mechanisms underlying inhibition deficits in children with comorbidities are more complex compared to those in the other two groups with singular disorders. It can be deduced that the attention problems in ASD+ADHD may differ in etiology from those in ADHD. 

### 4.1. Implications for Intervention

If excitatory neurotransmitter deficiency is not inherent in children with ASD+ADHD, the supplementation of psychostimulants may not necessarily improve attention problems. Instead, it might exacerbate their emotional issues [9]. This may explain why individuals with ADHD respond well to methylphenidate (MPH) and other psychostimulants, contrasting with the comparatively less effective outcomes observed in individuals with ASD+ADHD. Additionally, they exhibit a higher susceptibility to adverse effects like anxiety and irritability [8,50]. Yet, a notable proportion of individuals diagnosed with ASD+ADHD also show positive responses to stimulant medications [51]. Due to the substantial variability among individuals within the ASD+ADHD population, coupled with the interaction between ASD and ADHD, medication efficacy varies among different individuals. Therefore, treatment for children with ASD+ADHD should be more individualized. SP characteristics can serve as a starting point to help us better understand the sensory response traits of different individuals, speculate on emotional issues, prescribe medication accurately, and minimize the occurrence of side effects. Interventions for individuals with ASD+ADHD should not consist solely of ASD interventions combined with ADHD treatments. Besides exercising caution with medication, it is also essential to pay close attention to emotional issues often co-occurring in this population [52]. Future research could delve deeper into the precise influence of sensory systems on the EF of children with ASD+ADHD, offering insights for tailored interventions.

### 4.2. Limitations

Initially, SP-2 and Brief-2 data were obtained from parent questionnaires rather than through direct observation, aligning with the typical approach in the published studies. It is acknowledged that parental assessments could potentially introduce bias into the findings. Integrating experimental paradigms into future research could enhance the objectivity of the findings. Secondly, our sample size is relatively small, and there is a predominance of male participants, although the gender ratio in this study is similar to that in clinical studies of the other three neurodevelopmental disorders. Hence, the implications of this study may primarily extend to male children with ASD. Future studies could expand the sample size and enhance female representation to further assess potential gender differences. Finally, the conclusions drawn in this study may not be generalizable to the wider population, since it specifically focused on high-functioning children with ASD, ASD+ADHD, and ADHD, all with an FSIQ above 70. Nevertheless, with comparable levels of non-verbal intellectual functioning observed across the three neurodevelopmental disorder groups, direct comparisons between them are feasible. Future research could incorporate children with intellectual disabilities to diversify and enhance the generalizability of the study.

## 5. Conclusions

This study assessed SP, EF, and their correlation in children with ASD, ADHD, ASD+ADHD, and TD children. Children with ASD+ADHD exhibit more atypical SP and more severe EF deficits. The heightened sensory over-responsivity and the negative self-regulation in children with ASD+ADHD potentially play a role in exacerbating emotional regulation difficulties in comorbid children. Nevertheless, in contrast to children with ADHD, SP in children with ASD+ADHD does not serve as a predictor for inhibitory function; thus, we draw the conclusion that the mechanisms underlying attention issues in ASD+ADHD may be distinct from those in ADHD alone, highlighting the necessity for more customized therapeutic strategies. The findings from this study might shed light on the underlying causes of ASD+ADHD and potential strategies for tailored interventions.

## Figures and Tables

**Table 1:** Participant characteristics by group.

	ASD (N = 32)	ASD+ADHD (N = 30)	ADHD (N = 28)	TD (N = 30)	*p* Value	Post Hoc
Sex (male:female)	25:7	24:6	24:4	19:11	0.2177	
Age (SD), years	9.71 (2.12)	10.55 (2.10)	9.73 (1.33)	10.07 (1.65)	0.3135	
Non-verbal IQ	101.88 (13.65)	104.40 (14.11)	106.14 (12.11)	113.37 (8.97)	0.0033	TD > ASD, ASD+ADHD
SRS (raw score)	72.47 (19.51)	93.75 (22.43)	55.17 (20.78)	25.62 (13.20)	0.0001	ASD+ADHD > ASD > ADHD > TD

ASD: autism spectrum disorder; ADHD: attention-deficit hyperactivity disorder; ASD+ADHD: comorbidity form; TD: typically developing.

**Table 2:** Group comparisons for SP-2 and BRIEF-2, displayed for ASD, ASD+ADHD, ADHD, and TD.

	ASD (N = 32)	ASD+ADHD (N = 30)	ADHD (N = 28)	TD (N = 30)	*p* Value	Post Hoc
SP-2						
Seeking	38.44 (9.30)	45.93 (12.49)	44.89 (11.65)	31.40 (8.24)	0.0001	ASD+ADHD > ASD; ALL > TD
Avoiding	49.25 (10.55)	58.93 (12.84)	46.93 (10.81)	28.10 (8.53)	0.0001	ASD+ADHD > ASD, ADHD; ALL > TD
Sensitivity	39.41 (10.12)	47.57 (11.79)	43.89 (11.42)	27.86 (5.51)	0.0001	ALL > TD
Registration	47.50 (12.76)	60.00 (14.38)	48.79 (11.17)	31.97 (6.39)	0.0001	ASD+ADHD > ASD; ALL > TD
BRIEF-2 (T-Score)						
Inhibit	57.28 (9.90)	65.87 (9.35)	55.89 (9.67)	43.13 (5.39)	0.0001	ASD+ADHD > ASD, ADHD; ALL > TD
Shift	58.31 (7.99)	64.93 (10.36)	54.54 (8.78)	46.00 (5.45)	0.0001	ASD+ADHD > ASD, ADHD; ALL > TD
Index						
BRI	60.78 (9.93)	69.33 (8.08)	57.68 (9.81)	44.40 (5.70)	0.0001	ASD+ADHD > ASD, ADHD; ALL > TD
ERI	56.13 (8.24)	63.9 (9.17)	55.93 (9.71)	46.10 (4.89)	0.0001	ASD+ADHD > ASD, ADHD; ALL > TD
CRI	59.25 (8.30)	68.47 (7.26)	65.93 (6.09)	48.17 (5.55)	0.0001	ADHD, ASD+ADHD > ASD; ALL > TD
GEC	60.66 (8.37)	71.03 (8.51)	64.96 (7.50)	47.03 (5.53)	0.0001	ASD+ADHD > ADHD, ASD; ALL > TD

ASD: autism spectrum disorder; ADHD: attention-deficit hyperactivity disorder; ASD+ADHD: comorbidity form; TD: typically developing. ALL: all other groups; BRI: Behavior Regulation Index; ERI: Emotion Regulation Index; CRI: Cognitive Regulation Index; GEC: Global Executive Composite.

**Table 3:** Correlations between BRIEF-2 and SP-2 subscale scores within the ASD group.

ASD Group	Seeking	Avoiding	Sensitivity	Registration
Inhibit	0.331	0.496 **	0.375 *	0.392 *
Shift	0.21	0.430 *	0.261	0.194
BRI	0.354 *	0.514 **	0.462 **	0.433 *
ERI	0.293	0.558 ***	0.374 *	0.288
CRI	0.274	0.485 **	0.612 ***	0.480 **
GEC	0.336	0.529 **	0.541 ***	0.447 *

ASD: autism spectrum disorder; BRI: Behavior Regulation Index; ERI: Emotion Regulation Index; CRI: Cognitive Regulation Index; GEC: Global Executive Composite. * *p* ≤ 0.05; ** *p* ≤ 0.01; *** *p* ≤ 0.001.

**Table 4:** Correlations between BRIEF-2 and SP-2 subscale scores within the ASD+ADHD group.

ASD+ADHD	Seeking	Avoiding	Sensitivity	Registration
Inhibit	0.433 *	0.406 *	0.25	0.387 *
Shift	0.147	0.591 ***	0.530 **	0.589 ***
BRI	0.382 *	0.452 *	0.313	0.456 *
ERI	0.15	0.717 ***	0.508 **	0.553 **
CRI	0.138	0.418 *	0.355	0.390 *
GEC	0.254	0.602 ***	0.553 **	0.593 ***

ASD+ADHD: comorbidity form; BRI: Behavior Regulation Index; ERI: Emotion Regulation Index; CRI: Cognitive Regulation Index; GEC: Global Executive Composite. * *p* ≤ 0.05; ** *p* ≤ 0.01; *** *p* ≤ 0.001.

**Table 5:** Correlations between BRIEF-2 and SP-2 subscale scores within the ADHD group.

ADHD Group	Seeking	Avoiding	Sensitivity	Registration
Inhibit	0.638 ***	0.437 *	0.491 **	0.472 *
Shift	0.294	0.472 *	0.273	0.224
BRI	0.595 ***	0.465 *	0.373	0.464 *
ERI	0.351	0.490 **	0.225	0.130
CRI	0.354	0.465 *	0.491 **	0.395 *
GEC	0.567 **	0.570 **	0.434 *	0.417 *

ADHD: attention-deficit hyperactivity disorder; BRI: Behavior Regulation Index; ERI: Emotion Regulation Index; CRI: Cognitive Regulation Index; GEC: Global Executive Composite. * *p* ≤ 0.05; ** *p* ≤ 0.01; *** *p* ≤ 0.001.

**Table 6:** Regression models for SP-2 quadrant and BRIEF-2 Indices.

Item	Group	EF/Non-Verbal IQ	Ajusted R2	B	SEB	β	*p*-Value
BRI	ASD	Avoiding	0.282	0.544	0.165	0.578	0.003 **
		Non-Verbal IQ		−0.059	0.115	−0.082	0.61
	ADHD	Seeking	0.445	0.604	0.124	0.717	<0.001 ***
		Non-Verbal IQ		0.009	0.123	0.011	0.944
ERI	ASD	Avoiding	0.364	0.459	0.129	0.587	0.001**
		Non-Verbal IQ		−0.008	0.09	−0.013	0.933
	ASD+ADHD	Avoiding	0.502	0.542	0.101	0.759	<0.001 ***
		Non-verbal IQ		0.088	0.094	0.136	0.358
CRI	ASD	Sensitivity	0.448	0.441	0.119	0.538	<0.001 ***
		Non-Verbal IQ		−0.132	0.084	−0.217	0.126
Shift	ASD+ADHD	Avoiding	0.281	0.514	0.137	0.637	<0.001 ***
		Non-Verbal IQ		0.068	0.128	0.092	0.602
Inhibit	ADHD	Seeking	0.431	0.607	0.124	0.732	<0.001 ***
		Non-Verbal IQ		−0.026	0.123	−0.032	0.835
	ASD	Avoiding	0.321	0.543	0.16	0.578	0.002
		Non-Verbal IQ		−0.007	0.111	−0.01	0.949

ASD: autism spectrum disorder; ADHD: attention-deficit hyperactivity disorder; ASD+ADHD: comorbidity form; RI: Behavior Regulation Index; ERI: Emotion Regulation Index; CRI: Cognitive Regulation Index. ** *p* ≤ 0.01; *** *p* ≤ 0.001.

## Data Availability

The datasets used and analyzed during the current study are available from the corresponding author upon reasonable request.
